# Metacognitive ability predicts learning cue-stimulus associations in the absence of external feedback

**DOI:** 10.1038/s41598-018-23936-9

**Published:** 2018-04-04

**Authors:** Marine Hainguerlot, Jean-Christophe Vergnaud, Vincent de Gardelle

**Affiliations:** 1 Centre d’Economie de la Sorbonne, CNRS UMR 8174 Paris, France; 2 CNRS and Paris School of Economics, Paris, France

## Abstract

Learning how certain cues in our environment predict specific states of nature is an essential ability for survival. However learning typically requires external feedback, which is not always available in everyday life. One potential substitute for external feedback could be to use the confidence we have in our decisions. Under this hypothesis, if no external feedback is available, then the agents’ ability to learn about predictive cues should increase with the quality of their confidence judgments (i.e. metacognitive efficiency). We tested and confirmed this novel prediction in an experimental study using a perceptual decision task. We evaluated in separate sessions the metacognitive abilities of participants (N = 65) and their abilities to learn about predictive cues. As predicted, participants with greater metacognitive abilities learned more about the cues. Knowledge of the cues improved accuracy in the perceptual task. Our results provide strong evidence that confidence plays an active role in improving learning and performance.

## Introduction

Developing knowledge about one’s own environment, e.g. learning how environmental cues predict the occurrence of future stimuli or rewards, is essential to optimize our behavior. Past research has shown that such (probabilistic) associations can be learned, for instance using reinforcement mechanisms^1,2^ or Bayesian inference^3,4^. Typically, these learning mechanisms require external feedback. How agents learn when external feedback is unavailable remains, however, unclear. This is highly problematic given that one may easily consider situations in which agents make repeated decisions but receive feedback only after a long delay, if at all. For instance, a radiologist inspecting mammograms for potential tumors can only obtain decisive feedback on a case via surgery. In such situations, can the decision makers learn about potential predictive cues (e.g. environmental risk factors such as chemical exposure) that might help improve their decisions?

One intriguing hypothesis is that decision confidence could be useful in such situations. In support of this hypothesis, several recent studies offer evidence that reinforcement learning can be driven by confidence when external feedback is absent, in particular via midbrain dopamine signals representing the confidence “prediction error” involved in the learning process^5,6^. Here, we develop and test another empirical prediction on the basis of this hypothesis. Specifically, we reasoned that if confidence plays the role of feedback to guide learning about cue-stimulus associations, then successful learning (that is, the ability of the agent to identify the predictive value of a cue) should be positively related to the quality of the agent’s confidence judgments (that is, the ability of the agent to distinguish between correct responses and errors).

For example, let’s consider that our radiologist examines two groups of patients, one group who has been exposed to a particular chemical product that increases the risk of having cancer, and one group who has not been exposed to this product. How can the radiologist infer that the chemical exposure is a risk factor for cancer? We can identify at least two factors that would help the radiologist. The first factor is decision accuracy: if decisions are correct, there should be an increase in cancer detection in the exposed group, which the radiologist could eventually notice. The second factor is the quality of the beliefs that the observer has about the stimuli. This factor can be evaluated separately, through the quality of confidence judgments, that is, the extent to which confidence judgments are predictive of decision accuracy. We present a formalized account of these two factors, with a mathematical argument, in the Supplementary Materials.

To understand why the quality of confidence judgments can help to learn in the absence of external feedback, let’s consider the most extreme cases. If the radiologist can produce perfect confidence judgments, she would assign high confidence to her correct diagnostics and low confidence to her incorrect diagnostics. In a way, the radiologist now has perfect information about the presence or absence of the tumor, which she can link to the presence or absence of chemical exposure, so as to learn whether exposure increases the risk of breast cancer or not. On the other hand, if the radiologist’s confidence judgments poorly discriminate between correct and incorrect diagnostics, i.e. if confidence does not bear any information about diagnostic accuracy, then learning cannot exceed what is possible from the accuracy of decisions.

In other words, we predict that successful identification of an environmental cue should depend on the agent’s ability to form accurate beliefs about the cases, i.e. the agent’s ability to form confidence judgments about her own decisions, while controlling for the agent’s decision accuracy. We note that the alternative hypothesis is that learning is only based on responses and not beliefs, in which case learning should depend on decision accuracy but it should be unrelated to confidence.

We set out to test this prediction in a laboratory experiment. To do so, we engaged participants in a difficult perceptual task, which served as a basis for us to evaluate their abilities to form confidence judgments and their abilities to identify predictive environmental cues, in two distinct sessions. As explained above, if confidence in decision is used as a substitute for external feedback, then being able to distinguish between one’s correct and incorrect decisions should be key in identifying the cues. In other words, if our hypothesis is true, the assessment of metacognitive abilities in one experimental session should predict whether or not participants will learn about the cues in the other experimental session. Furthermore, our experiment also allowed us to explore whether the identification of cue-stimulus associations eventually influenced participants’ behavior in the perceptual task.

**Figure 1:**
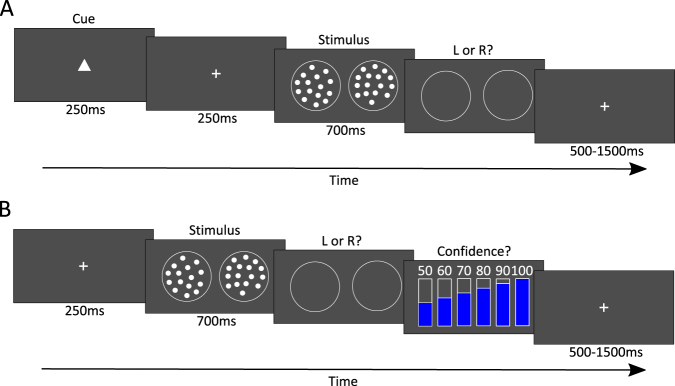
Experimental Design. In both sessions, participants performed the same perceptual task. On each trial, they had to decide which circle (left or right) contained more dots. (**A**) Learning session. A cue in the form of a geometric shape (a square, circle or triangle) was presented before the stimulus. Two cues were respectively predictive of the left and right circle whereas one cue was non- predictive. Participants were not informed about the associations between these cues and the stimulus categories. At the end of the session, they had to identify the cue-stimuli associations. (**B**) Confidence Session. Participants gave a confidence rating after each decision.

Our perceptual task was as follows: on each trial, participants indicated which of two circles, the left or right, contained more dots (Fig. 1). They received no feedback. Two experimental sessions were taken, in a counterbalanced order across participants. Both sessions started with a working memory test and an initial calibration phase for the perceptual task (for details see the Methods section).

In the “learning session” (600 trials), a geometric shape (circle, square or diamond) preceded the stimulus. One shape predicted the left category, one predicted the right category (both with probability p = 0.75) and one provided no information about the forthcoming category. We informed participants that there were a ‘left’, a ‘right’ and a ‘neutral’ cue but without specifying the probability of the predictive cue. We instructed participants to learn and use the associations between cues and categories, so as to maximize their performance during the task. At the beginning of the learning session, we told participants that they would have to identify these cue-stimuli associations at the end of the session. In our result section, we shall define “learners” and “non-learners” on the basis of whether participants indeed successfully identified the associations between the cues and the stimuli.

In the “confidence session” (512 trials), participants gave a confidence rating after each decision. Confidence quality was measured as the metacognitive efficiency^7^, that is, how good one is at distinguishing between correct and incorrect decisions, after controlling for perceptual performance. Our idea is that metacognitive efficiency is a proxy for the ability of participants to formalize their beliefs about the stimuli, and that this ability is roughly constant across sessions. Thus, under our hypothesis, we predict that metacognitive efficiency should be positively related to the successful identification of the cue-stimuli associations.”

## Results

Our analysis proceeds in 2 steps. First, we tested our hypothesis that metacognitive efficiency predicted learning, by which we mean the successful identification of the cues by participants. Second, we assessed whether this identification influenced participants’ cue usage.

### From metacognitive efficiency to successful cue identification

**Figure 2:**
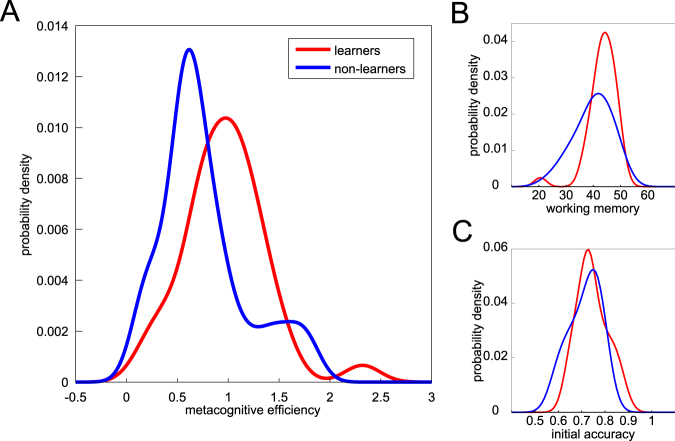
Predicting cue identification. (**A**) Distributions of the metacognitive efficiency (ratio of meta-d’ over d’) across participants who successfully identified the 3 cue-stimuli associations (“learners”), and participants who did not (“non-learners”). (**B**) Distributions of working memory scores for “learners” and “non-learners”. (**C**) Distributions of the initial accuracy in the learning session, for “learners” and “non-learners”.

Our main hypothesis related metacognitive efficiency to successful cue identification, which was coded as 1 for participants who correctly classified the 3 cue-stimuli associations (N = 35) and 0 (N = 30) for participants who identified either 1 (N = 27) or 0 association (N = 3). Metacognitive efficiency was quantified as the ratio of meta-d’ over d’ for each individual (M = 0.87, SD = 0.42). Critically, we found that our main hypothesis was confirmed: metacognitive efficiency was higher in participants who successfully identified the cue-stimuli associations (“learners”: M = 0.972, SD = 0.405) than for participants who did not (“non-learners”: M = 0.758, SD = 0.424), as illustrated in Fig. 2A. The difference in metacognitive efficiency between “learners” and “non-learners” was significant (T-test: t(63) = −2.078, p = 0.0418).

We conducted several robustness checks to ensure that this difference between “learners” and “non-learners” was not due to some specific features of the metacognitive measure we chose. Firstly, we also found this difference to be significant when we used the log ratio of meta d’ over d’ instead of the ratio (T-test: t(63) = −2.261, p = 0.0272), or when we used a rank test instead of a classic t-test (Rank sum test: z = −2.533, p = 0.0113). Secondly, given that the computation of metacognitive efficiency as the ratio of meta-d’ over d’ can be problematic when participants do not display a lot of variance in their confidence judgments, we verified that the difference between “learners” and “non-learners” was still significant after excluding 7 participants who used the same confidence level in more than 80% of the trials (T-test: t(56) = 2.744, p = 0.008; Rank sum test: z = 3.072, p = 0.002). Moreover, we replicated this difference between “learners” and “non-learners” with other measures of metacognitive abilities. The resolution score from the Brier index was higher in participants who successfully learned the cue-stimuli associations compared to participants who did not (M = 0.016, SD = 0.010; M = 0.010, SD = 0.009; T-test: t(63) = −2.413, p = 0.0188; Rank sum test: z = −2.559, p = 0.0105). The difference between the average confidence in correct responses and average confidence in incorrect responses was also greater for “learners” than for “non-learners” (M = 0.089, SD = 0.063; M = 0.058, SD = 0.049; T-test: t(63) = −2.197, p = 0.0317; Rank sum test: z = −2.323, p = 0.0202). These analyses thus confirmed the hypothesized relation between metacognitive efficiency and cue identification.

To ensure that this result was not simply due to inter-individual differences in motivation, perceptual abilities or memory abilities, we conducted a multiple logit regression analysis in which successful cue identification was predicted from metacognitive efficiency along with additional predictors coding for these factors. In short, although memory and perceptual abilities did facilitate cue identification, metacognitive efficiency still predicted cue identification when we simultaneously controlled for these confounds (β = 1.805, se = 0.777, t = 2.324, p = 0.020). We report below the full results of this regression (see also Table S1 of the Supplementary Materials).

Memory abilities of participants were evaluated using a separate memory test (see Methods) and exhibited a large variability across participants (M = 41.7, SD = 6.2). We found that greater memory scores were associated with successful cue identification in our regression (β = 0.133, se = 0.059, t = 2.271, p = 0.023). Figure 2B also illustrates how memory scores were greater for “learners” than for “non-learners” (T-test: t(63) = −2.295, p = 0.0251). This is expected given that working memory is needed to learn: in order to update her estimation of a cue-stimulus association, the participant needs to remember both the cue presented at the beginning of the trial and her previous estimate of the cue-stimulus associations for this cue.

To control for perceptual abilities in the task, we also included in our regression a predictor coding for the accuracy in the perceptual task at the beginning of the learning session. Indeed, although we attempted to calibrate performance at 71% before the experiment, this initial accuracy still varied across participants (M = 73%, SD = 6%). We expected this initial accuracy to be predictive of successful learning of the cues, since any advantage in the perceptual task would increase the amount of information on which learning can be based (for an illustration see the Supplementary Materials). We thus defined initial accuracy as the proportion of correct responses over trials 1–96 (we have checked that our results are unchanged if we use 48, 72 or 120 trials instead of 96). Our multivariate regression confirmed that initial accuracy significantly increased the probability of learning the cues (β = 11.812, se = 5.645, t = 2.092, p = 0.036). Figure 2C illustrates how, although the modes of the two distributions appear to be roughly the same, the range of initial accuracy is shifted for “learners” compared to “non-learners”, resulting in a difference between the two groups (T-test: t(63) = 2.029, p = 0.0467).

Finally, to control for motivation to do well in the task, we introduced three additional predictors in our regression: one coding for calibrated difference in the number of dots (which would be lower for more motivated participants), one coding for the order in which the sessions were performed (confidence or learning session first) and one coding for the interaction between metacognitive efficiency and this session order. None of these predictors had an effect on successful learning (all p > 0.49).

### From cue identification to cue usage

Our next series of analysis focused on the impact of cue identification on performance in the task. Overall, “learners” exhibited better performance in the task compared to “non-learners” (M = 0.754, SD = 0.055 vs. M = 0.704, SD = 0.045; t(63) = 4.001, p = 0.0002). However, the direction of the influence remains unclear in this analysis: cue identification might lead to an increase in performance, but performance should also help for cue identification, as we explained in the previous section.

**Figure 3:**
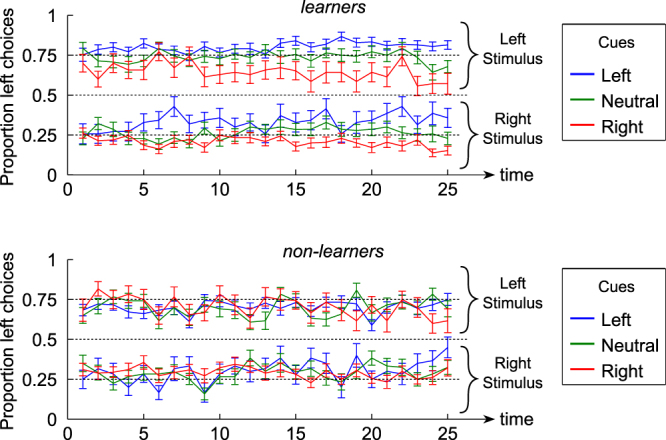
Timecourse of the cue influence. The proportion of left choices is plotted for each stimulus and cue, as a function of time, separately for “learners” and “non-learners” participants. Each point in time represents a block of 8 trials. Error bars represent the mean and the standard error of the mean across participants, in each group.

To better isolate the effect of cue identification on performance, we thus focused on how participants’ responses followed the information provided by the right and left predictive cues. We found that responses of “learners” were more congruent with the cues than responses of “non-learners” (M = 0.684, SD = 0.059 vs. M = 0.602, SD = 0.059; t(63) = 5.604, p < 0.001). Furthermore, comparing response accuracy when the cue was valid (i.e. in 75% of the trials) to when the cue was invalid (in 25% of the trials), we found that “learners” were indeed more likely to be correct when the cue was valid than when the cue was invalid (valid: M = 0.798, SD = 0.065; invalid: M = 0.657, SD = 0.114; t(34) = 6.109, p < 0.001). By contrast, accuracy was not modulated by cue validity for “non-learners” (valid: M = 0.705, SD = 0.070; invalid: M = 0.702, SD = 0.080; t(29) = 0.136, p = 0.89). The effect of cue validity on accuracy was significantly different between the two groups (t(63) = 4.418, p < 0.001). In other words, not only have “learners” identified that the cue provided valuable information about the stimulus, but they also used this information to increase their performance (see also Table S2). Figure 3 further illustrates how perceptual decisions evolved over time for “learners” and “non-learners”, as a function of the stimulus presented and the cue. For each stimulus there are thus 3 curves, corresponding to the 3 cues, which get separated as time passes, for “learners” but not for “non-learners”.

To further investigate how “learners” and “non-learners” differed in their use of the cue, we conducted an analysis based on Signal Detection Theory (Green & Swets, 1966) to separate perceptual sensitivity and decision criterion. In particular, we evaluated the difference between the decision criteria adopted for the cues that predicted left and right responses. Critically, this criterion difference was significant for “learners” (t(34) = 5.991, p < 0.001) but not for “non-learners” (t(29) = 0.061, p = 0.9520), resulting in a significant difference between the two groups (t(63) = 4.479, p < 0.001). For completeness, we also looked at perceptual sensitivity (d’, averaged across all 3 cues), and found that it was also higher for “learners” compared to “non-learners” (M = 1.334, SD = 0.436 vs. M = 1.113, SD = 0.302; t(63) = 2.332, p = 0.0229). This may reflect the fact that perceptual sensitivity provides information that can be used to learn about the cues, such that participants with greater sensitivity are more likely to end up as “learners” compared to participants with lower sensitivity.

As a final step, we investigated whether metacognitive efficiency directly predicted cue usage across participants. The correlations between metacognitive efficiency and average performance (r = −0.020, p = 0.877) or congruency with the cue (r = −0.027, p = 0.828) were not significant. However, we could find an effect of metacognitive efficiency on performance when we controlled for inter-individual differences in memory, perceptual abilities and motivation. To do so, we conducted a multiple linear regression analysis (similar to the one in the previous section) in which final performance (here defined as the mean accuracy after trial 96 in the learning session) was predicted from metacognitive efficiency, along with memory scores, the calibrated difference in the number of dots, the session order, the interaction between metacognitive efficiency and session order, as well as initial performance in the learning session (mean accuracy before trial 96) that may capture inter-individual differences in perceptual abilities (possibly due to an imperfect calibration of the stimuli). We found, indeed, that final accuracy was significantly predicted by initial accuracy (β = 0.643, se = 0.076, t = 8.464, p < 0.001). More critically, final accuracy in the learning session was also positively affected by metacognitive efficiency (β = 0.027, se = 0.011, t = 2.530, p = 0.014). None of the other predictors were significant in this analysis. The full results of this regression are reported in Table S1 of Supplementary Materials. We note, in addition, that introducing criterion difference as yet another predictor in this multiple regression did not change these findings, as final accuracy remained significantly predicted only by metacognitive efficiency (β = 0.026, se = 0.010, t = 2.514, p = 0.015) and initial accuracy (β = 0.625, se = 0.076, t = 8.180, p < 0.001) in this regression.

### Discarding two alternative schemes linking cue identification, usage, and metacognitive efficiency

First, one question that arises when considering cue usage then is whether cue identification would be based solely on the adoption of different decision criteria for the predictive cues, without any direct effect of metacognitive efficiency. To evaluate this possibility we entered the criterion difference as another covariate in the multiple logistic regression reported in the previous section, in which metacognitive efficiency was used to predict successful cue identification, along with the covariates previously introduced (namely session order, interaction of metacognitive efficiency and session order, memory scores, calibrated difference in the number of dots, and initial accuracy). We found that although the criterion difference was a significant predictor of successful cue identification (β = −4.329, se = 1.391, t = −3.112, p = 0.0019), the effect of metacognitive efficiency remained significant (β = 1.846, se = 0.894, t = 2.065, p = 0.039). In other words, the effect of metacognitive efficiency on cue identification could not be fully explained by cue usage.

Second, one could envision that metacognitive efficiency may have no impact on learning during the task and may only help participants to introspect and evaluate their use of the cues at the end of the task, when asked to report the cue-stimulus associations. If so, participants with higher metacognitive efficiency would be better able to report the cue-stimulus associations that correspond to their cue usage. We tested this prediction in our data by looking at whether the ordering of the cues in their post-hoc identifications corresponded to the ordering of the cues in terms of cue usage (using the decision criteria calculated with Signal Detection Theory). We found 14 participants for whom the two orderings were indeed equal, and they did not have a better metacognitive efficiency than the other participants (T-test: t(63) = 0.0129, p = 0.99). In sum, metacognitive efficiency did not seem to increase the correspondence between cue usage and cue identification. Thus, our working hypothesis is that metacognitive efficiency helps participants to learn about the cues while they are doing the task.

## Discussion

Identifying cues that can help performing a difficult task is a useful ability, in a world where agents have to make decisions under uncertainty. Here, we show that inter-individual heterogeneity in the ability of participants to evaluate their own performance, measured in an independent session, could predict the successful classification of predictive cues. Importantly, this successful learning of predictive cues also translated into actual benefits in terms of task performance.

Our study was based on the hypothesis that confidence could be used as a substitute for external feedback signal when it is not available for learning about cues. To evaluate the quality of this confidence signal, we relied on the metacognitive efficiency of participants, as estimated from their confidence reports. Our results seem to confirm our hypothesis.

Our study relates in particular to two recent studies investigating the role of confidence in learning, although with different learning problems. One study used a category learning task: participants had to learn how perceptual stimuli defined in a two-dimensional space were mapped onto two category labels^5^. In this study, participants could learn the mappings from some ‘association’ trials in which exemplars were presented in conjunction with the correct category label, while on the ‘test’ trials they had to categorize the stimuli and give their confidence. The other study investigated a different form of learning, namely perceptual learning^6^. In this paradigm, participants were engaged in a perceptual categorization task, and they knew the mappings between responses and categories from the start (e.g. ‘your task is to press left if the stimulus is tilted clockwise relative to the reference’). Performance nevertheless improved over the course of the experiment, as if participants could learn to better extract the information from the stimuli. Our study implements yet another form of learning, as participants here received two types of information, a perceptual stimulus and a symbolic cue, and had to learn about the symbolic cue on the basis of their estimation of perceptual performance. Together, this set of studies thus provides support for the involvement of confidence in learning, in an increasing range of situations.

One other noticeable difference between the present study and the two aforementioned studies is that in these studies confidence judgments were collected during the learning sessions, while in our paradigm confidence was only evaluated from a distinct session taking place on a different day. This is interesting because it shows that confidence does not need to be explicitly evaluated and verbalized to be used for learning. These results echo previous research showing that confidence is evaluated automatically during decision-making^8^. We would also argue that since confidence was used to guide the evaluation of a symbolic cue, it should be readily available at an abstract level of representation^9,10^.

The present work was motivated by the hypothesis that confidence can be used to guide learning, when no external feedback is available. Our study provides support for this hypothesis, and extends past research along these lines, as discussed above. We believe nonetheless that many questions remain open regarding the ability of humans to learn from their own confidence. How fast can observers learn in this situation? Are observers able to evaluate the precision of what they learn, and to use this precision in optimal manner? These questions have been addressed in situations in which observers learn about event probabilities from the past history of events^11,12^. They remain unanswered in situations in which learning needs to be based on confidence.

In addition, one issue that remains to be further investigated is the nature of the signals that are used in what we described as a confidence-based learning. It is reasonable to expect that participants merely evaluate the strength of the evidence that they have extracted from the stimulus, but past research has also demonstrated that confidence reports about perceptual decisions might be influenced by other quantities as well, such as specific properties of the stimuli^13,14^, the current attentional state^15^, the past history of stimuli^16^, or the noise at the level of the motor response^17^. Participants might as well rely on simple heuristics such as detecting a lapse of attention, the occurrence of eye blinks, or of pressing the wrong key. Our reasoning did not hinge specifically on one or the other of these (not mutually exclusive) possible factors, which are also difficult to measure with our experimental data. Nevertheless, one interesting question for further research would be to evaluate the role of participants’ awareness of their attentional lapses. Indeed, such lapses could lead to an underestimation of the true perceptual sensitivity by the experimenter, but they could be captured by the participant’s confidence judgments, who might then exhibit a metacognitive efficiency score greater than one. Whether the link between learning (which also depends on perceptual sensitivity) and metacognitive efficiency we found is driven by such cases constitutes an open empirical question, which requires an independent measure of attentional lapses to be addressed.

The specific learning mechanism that might be at play also deserves more scrutiny. In our paradigm, participants might have updated their estimate of the value of the cue from their confidence in the current perceptual decision. Past studies^5,6^ have specifically put forward a particular form of confidence-based reinforcement learning, in which the prediction error on confidence would be used to update the synaptic weights between sensory detectors and decision units, so as to increase perceptual performance over time. Yet, alternative mechanisms could be considered as well. For instance, a reinforcement learning mechanism can achieve optimal inference if the learning rate, rather than being a constant, changes appropriately as a function of the estimated volatility of the environment. Humans seem able to do so when they learn from event history^4^. Whether this holds true in confidence-based learning without external feedback is an open empirical question. Do we know when to trust our own confidence? We believe that this issue opens exciting perspectives for further research.

## Methods

### Participants

65 individuals (28 females; mean ± SD age, 22.31 ± 3.12 years) were recruited through the Laboratory of Experimental Economics research pool in Paris (LEEP) and gave informed consent to participate. The study was conducted in line with the principles of the Declaration of Helsinki. Participants came to two sessions, which took place 4 days apart. The experiment was conducted with groups of 15–20 participants. Participants received 13 Euros for participating plus an incentivized bonus as described below.

### Ethics statement

Written informed consent was obtained from all participants before the experiment. The research was non-invasive; it involved negligible risks and no collection of nominative/identifying information or health information. Thus, ethics approval was not required under French regulations, and no IRB was consulted before conducting the study.

### Summary of the design

Our study involved a simple perceptual categorization task (see Fig. 1). On each trial, participants indicated which of two circles, the left or right, contained more dots. They received no feedback. Two experimental sessions were taken, in a counterbalanced order across participants. In the “confidence session” (512 trials), participants gave a confidence rating after each decision. Confidence quality was measured as the metacognitive efficiency^9^, that is, the information contained in confidence ratings about the stimuli, after controlling for perceptual performance. In the “learning session” (600 trials), a geometric shape (circle, square or diamond) preceded the stimulus. One shape predicted the left category, one predicted the right category (both with probability p = 0.75) and one provided no information about the forthcoming category. We instructed participants to learn and use the associations between cues and categories, so as to maximize their performance during the task. At the end of the session, participants also had to identify these cue-stimuli associations. Both sessions started with a working memory test and an initial calibration phase for the perceptual task.

### Perceptual Task

On each trial, after a 250 ms fixation cross, two sets of about 100 dots were simultaneously presented for 700 ms, one on the left side and one on the right side of the computer screen. Participants had to indicate which set contained more dots, by pressing the corresponding arrow on the keyboard. After the response, the inter-trial interval was jittered between 0.5 s and 1.5 s. Participants received no feedback about the accuracy of their decision. Response times shorter than 200 ms or longer than 2200 ms (from stimulus onset) were discouraged by presenting a “too fast” or “too slow” message on the screen. The experiment was run using MATLAB (MathWorks) and Psychotoolbox^18^, on screens (resolution 1024 × 768) viewed at normal distance (about 60 cm).

### Calibration

Stimulus difficulty was calibrated for each participant at the beginning of each session. Specifically, one circle contained 100 dots while the other circle (the stimulus) contained 100 + *x* dots. The other circle was computed separately for the right (${x}_{r}$) and left (${x}_{l}$) stimulus by adjusting with a 2-down 1-up rule^19^, to obtain 71% of “left” or “right” responses, in two interleaved staircases of 150 trials each. The step size of the staircases was reduced from 20 to 16, 8, 4 and 2 dots on trials 12, 24, 60 and 80 respectively. After the calibration phase, the dots difference ${x}_{r}$ and ${x}_{l}$ conditional on the right and left stimulus respectively was constant across the session.

### Confidence session

Each response was followed by a confidence rating, in which participants indicated their subjective belief that their response just given was correct, on a 6 steps scale ranging from 50% confident (i.e. guess) to 100% confident, in 6 steps of 10%. Participants responded using the numerical keys on the top-left of the keyboard. This confidence rating was incentivized using a probability matching rule^20^. The participant is offered an exchange between his response and a lottery ticket with a probability P of success. The number P is generated randomly on each trial, and compared to the confidence response. If P (the success probability) is greater than the confidence, then the participant’s reward is determined by the lottery. If not, it is determined by the accuracy of the response. The mechanism was presented to participants as a way to maximize their earnings by providing accurate confidence ratings. Instructions, examples, and a training phase with feedback (40 trials) were included to make sure that participants understood the mechanism. Participants then completed 512 trials in the session.

### Metacognitive efficiency measure

We estimated metacognitive sensitivity using the meta-d’ method^7^. In a Signal Detection framework, Meta-d’ corresponds to the level of type 1 SDT sensitivity (d’) that a metacognitively ideal observer would have needed to produce the observed type 2 data. Metacognitive efficiency is defined by the relative measure meta-d’/d’. If the ratio is equal to one, then the observer is metacognitively ideal. If meta-d’/d’ is lower than one, then the observer is metacognitively inefficient. It may also occur that the ratio takes values above one, for instance if additional information is used after the initial choice. Here, we applied the code as provided at http://www.columbia.edu/bsm2015/type2sdt/ with the default settings. In particular, we used the default assumption of “equal variance” between the two stimuli, and the default “cell padding” strategy to avoid empty cells in the confidence × response design, noting that 45 participants out of 65 had at least one empty cell out of 24 (6 ratings × 2 stimuli × 2 responses). We report in the supplementary material the values of meta-d’ along with the distributions of confidence ratings for each individual participant.

### Learning session

Each trial started with a central cue presented for 250 ms, before the fixation cross. The cue was a square, a circle or a triangle. One shape predicted the left category, one predicted the right category (both with probability p = 0.75) and one provided no information about the forthcoming category. Participants were not informed about the associations between the cues and the prior probabilities of occurrence of a stimulus but they were informed that there were a ‘left’, a ‘right’ and a ‘neutral’ cue. At the beginning of the learning session, they were required to learn about the cue-stimulus associations, in order to optimize their decisions and they were told that at the end of the session, they had to report these associations. Participants completed 600 trials of interleaved sequences of blocks of 8 trials per cue. For each sequence of 8 trials (“block”), the same cue was displayed prior seeing the stimuli. For each block, the predictive cue indicated, in a random order, the forthcoming stimulus correctly on 6 trials (75% valid cue) and the forthcoming stimulus incorrectly on 2 trials (25% invalid cue). Response accuracy was incentivized: participants won 1 point if correct and lost 1 point if incorrect. The value of 1 point was 0.02 €. In addition, participants were informed, at the beginning of the session, that they would be rewarded 2 Euros, 1 or 0 Euro for correctly reporting 3, 1 or none of the associations.

### Memory span task

The task consisted of twelve trials. On each trial, participants received a sequence of *m* + *n* letters and were required to report in the forward order the last *n* letters. The (m, n) combinations were randomly drawn without replacement with *m* = 0, 1, 2 and *n* = 3, 4, 5, 6. Letters were sampled with replacement from a pool (F, H, J, K, L, N, P, Q, R, S, T, Y). After an initial fixation cross of 300 ms, letters were presented successively for 300 ms on the center of a gray background screen and followed by a 2200 ms interval. At the end of the sequence, participants were asked to report the last *n* letters by clicking on the cells of a 4 × 3 grid displaying the 12 letters of the pool. One point was earned for each item reported in the correct serial position. For example, if participants were instructed to report “N P T”, they would gain 3 points for responding “N P T” but 0 point for responding “L N P”. The maximum score possible was 54 points. The task was programmed in JavaScript and administered to participants through the internet based software REGATE version 9.33. For each participant, we computed the average value of the working memory scores obtained in each session.

### Significance statement

When one has to learn new task, it is helpful to obtain clear feedback from an external source, in order to uncover the correct cues to make the best decisions. However, such feedback is not always available. One hypothesis is that when external feedback is absent, humans use their internal sense of confidence to guide their learning. We confirmed this hypothesis in a laboratory experiment, within the context of a simple perceptual task: participants who could better evaluate their confidence were also more likely to successfully learn the useful cues for the task. These results demonstrate that within a particular decision task, there is an intimate relationship between the ability to learn and the ability to evaluate one’s own abilities.

## Electronic supplementary material

Supplementary Material

Data description

Dataset 1

## References

[1] Sutton, R. S. & Barto, A. G. Reinforcement Learning. (MIT Press, 1998).

[2] Rescorla, R. & Wagner, A. In Classical Conditioning II: Current Research and Theory (eds Black, A. & Prokasy, W.) 64–99 (Appleton-Century-Crofts, 1972).

[3] Courville AC, Daw ND, Touretzky DS (2006). Bayesian theories of conditioning in a changing world. Trends Cogn. Sci..

[4] Behrens TE, Woolrich MW, Walton ME, Rushworth MF (2007). Learning the value of information in an uncertain world. Nat Neurosci.

[5] Daniel R, Pollmann S (2012). Striatal activations signal prediction errors on confidence in the absence of external feedback. NeuroImage.

[6] Guggenmos, M., Wilbertz, G., Hebart, M. N. & Sterzer, P. Mesolimbic confidence signals guide perceptual learning in the absence of external feedback. eLife **5** (2016). 10.7554/eLife.13388 PMC4821804 27021283

[7] Maniscalco B, Lau H (2012). A signal detection theoretic approach for estimating metacognitive sensitivity from confidence ratings. Conscious. Cogn..

[8] Lebreton M, Abitbol R, Daunizeau J, Pessiglione M (2015). Automatic integration of confidence in the brain valuation signal. Nat Neurosci.

[9] de Gardelle V, Mamassian P (2014). Does Confidence Use a Common Currency Across Two Visual Tasks?. Psychol. Sci..

[10] de Gardelle V, Le Corre F, Mamassian P (2016). Confidence as a Common Currency between Vision and Audition. PLOS ONE.

[11] Meyniel F, Schlunegger D, Dehaene S (2015). The Sense of Confidence during Probabilistic Learning: A Normative Account. PLOS Comput. Biol..

[12] Meyniel F, Dehaene S (2017). Brain networks for confidence weighting and hierarchical inference during probabilistic learning. Proc. Natl. Acad. Sci..

[13] de Gardelle V, Mamassian P (2015). Weighting Mean and Variability during Confidence Judgments. PLOS ONE.

[14] Boldt A, de Gardelle V, Yeung N (2017). The Impact of Evidence Reliability on Sensitivity and Bias in Decision Confidence. J Exp Psychol Hum Percept Perform.

[15] Samaha J, Iemi L, Postle BR (2017). Prestimulus alpha-band power biases visual discrimination confidence, but not accuracy. Conscious Cogn.

[16] Rahnev D, Koizumi A, McCurdy LY, D’Esposito M, Lau H (2015). Confidence Leak in Perceptual Decision Making. Psychol Sci.

[17] Fleming SM (2015). Action-specific disruption of perceptual confidence. Psychol Sci.

[18] Brainard DH (1997). The Psychophysics Toolbox. Spat. Vis..

[19] Levitt H (1971). Transformed up-down methods in psychoacoustics. J. Acoust. Soc. Am..

[20] Massoni, S., Gajdos, T. & Vergnaud, J.-C. Confidence measurement in the light of signal detection theory. Front. Psychol. **5** (2014). 10.3389/fpsyg.2014.01455 PMC4263084 25566135

