# Supplementary material for: Metacognitive ability predicts learning cue-stimulus associations in the absence of external feedback
Source: Sci Rep. 2018 Apr 4;8:5602. doi: 10.1038/s41598-018-23936-9 (PMC5884814; doi:10.1038/s41598-018-23936-9)
Supplement: Supplementary file 1 — Supplementary Material [file 41598_2018_23936_MOESM1_ESM.docx]

Supplementary Online Material for:

**Metacognitive ability predicts learning cue-stimulus associations in the absence of external feedback**

Marine Hainguerlot^1^, Jean-Christophe Vergnaud^1,*^, Vincent de Gardelle^2,*^

^1^Centre d’Economie de la Sorbonne, CNRS UMR 8174, Paris, France

^2^CNRS and Paris School of Economics, Paris, France.

^*^denotes equal contribution

Corresponding author: Vincent de Gardelle.

Centre d’Economie de la Sorbonne, 112 boulevard de l’Hopital, 75013 Paris, France

Tel: 0144078742. Email: [vincent.gardelle@gmail.com](mailto:vincent.gardelle@gmail.com)

This file includes the following items:

- 3 Supplementary Tables (Table S1, Table S2 and Table S3)
- 1 Supplementary Figure (Figure S1)
- 1 Supplementary Analysis (‘Mathematical argument’)

|  | 1. Logit   Cue identification | | | (2) OLS  Final accuracy | | |
| --- | --- | --- | --- | --- | --- | --- |
|  | *b* | *se* | *p* | *b* | *se* | *p* |
| Metacog | 1.805 | 0.777 | 0.020 | 0.027 | 0.011 | 0.014 |
| W. Memory | 0.133 | 0.059 | 0.023 | 0.001 | 0.001 | 0.172 |
| Initial accuracy | 11.812 | 5.645 | 0.036 | 0.643 | 0.076 | <0.001 |
| Calibrated dots | 0.074 | 0.107 | 0.490 | 0.003 | 0.002 | 0.077 |
| Order | -0.034 | 0.590 | 0.954 | 0.013 | 0.009 | 0.152 |
| Metacog*Order | -0.002 | 0.305 | 0.996 | 0.006 | 0.004 | 0.176 |

**Table S1:** Multivariate regression results. (1) Logit regression with successful cue identification as a dependent variable. (2) OLS regression with final performance as a dependent variable. Explanatory variable: metacognitive efficiency. Control variables: working memory, initial accuracy, calibrated difference in the number of dots, order in which the sessions where performed (=1 if confidence session first, 0 otherwise) and interaction term between metacognitive efficiency and session order.

|  |  | Response rate left | Accuracy,  All trials | Accuracy, valid cue trials | Accuracy, invalid cue trials | SDT estimates | |
| --- | --- | --- | --- | --- | --- | --- | --- |
|  |  |  |  |  |  | c | d’ |
| « Learners » | Neutral cue | 0.502  (0.071) | 0.737  (0.068) | - | - | 0.004  (0.230) | 1.340  (0.479) |
|  | Left cue | 0.685  (0.076) | 0.767  (0.062) | 0.801  (0.081) | 0.665  (0.138) | 0.215  (0.287) | 1.354  (0.488) |
|  | Right cue | 0.316  (0.093) | 0.758  (0.063) | 0.795  (0.096) | 0.648  (0.132) | -0.240  (0.361) | 1.308  (0.431) |
| « Non- learners » | Neutal cue | 0.492  (0.107) | 0.703  (0.050) | - | - | -0.022  (0.333) | 1.126  (0.325) |
|  | Left cue | 0.598  (0.095) | 0.701  (0.063) | 0.701  (0.100) | 0.700  (0.117) | -0.010  (0.291) | 1.095  (0.324) |
|  | Right cue | 0.393  (0.081) | 0.707  (0.069) | 0.708 (0.095) | 0.704  (0.110) | -0.014  (0.246) | 1.117  (0.388) |

**Table S2:** Descriptive statistics on cue usage for “learners” (N=35) and “non- learners” (N=30). Response rate left, Average accuracy rate, accuracy rate conditional on valid and invalid cues, and SDT estimates decision criterion (c) and sensitivity (*d’*); with standard deviation reported between parentheses.

|  | Mean (SD) | (1) | (2) | (3) | (4) | (5) | (6) | (7) | (8) | (9) | (10) | (11) | (12) | (13) | (14) | (15) | (16) |
| --- | --- | --- | --- | --- | --- | --- | --- | --- | --- | --- | --- | --- | --- | --- | --- | --- | --- |
| (1) Cue learn | +0.54 (0.50) | 1 | +0.25 * | +0.27 * | +0.29 * | +0.28 * | +0.11 | -0.05 | +0.25 * | +0.45 *** | +0.48 *** | +0.58 *** | +0.58 *** | -0.22 | +0.49 *** | +0.28 * | +0.13 |
| (2) Metaratio | +0.87 (0.42) |  | 1 | +0.93 *** | +0.65 *** | +0.12 | -0.07 | -0.10 | -0.21 | -0.02 | +0.02 | -0.03 | -0.03 | +0.01 | -0.03 | -0.02 | -0.10 |
| (3) log(metaratio) | -0.27 (0.57) |  |  | 1 | +0.63 *** | +0.10 | -0.04 | -0.06 | -0.15 | +0.05 | +0.09 | -0.02 | +0.01 | +0.07 | -0.04 | +0.05 | -0.03 |
| (4) Brier Resol. | +0.01 (0.01) |  |  |  | 1 | +0.17 | -0.09 | -0.09 | -0.03 | +0.13 | +0.16 | +0.02 | +0.05 | +0.07 | -0.01 | +0.14 | +0.22 |
| (5) W. memory | +41.72 (6.21) |  |  |  |  | 1 | -0.33 ** | +0.06 | -0.08 | +0.01 | +0.03 | +0.06 | +0.02 | -0.09 | +0.05 | -0.01 | +0.09 |
| (6) Cal. Dots | +9.95 (3.36) |  |  |  |  |  | 1 | -0.24 | +0.47 *** | +0.47 *** | +0.45 *** | +0.26 * | +0.40 *** | +0.23 | +0.14 | +0.46 *** | +0.19 |
| (7) Order | +0.54 (0.50) |  |  |  |  |  |  | 1 | -0.06 | +0.00 | +0.02 | +0.06 | +0.02 | -0.12 | +0.09 | -0.04 | -0.08 |
| (8) Initial perf. | +0.73 (0.07) |  |  |  |  |  |  |  | 1 | +0.84 *** | +0.77 *** | +0.46 *** | +0.67 *** | +0.32 ** | +0.21 | +0.77 *** | +0.11 |
| (9) Overall perf. | +0.73 (0.06) |  |  |  |  |  |  |  |  | 1 | +0.99 *** | +0.59 *** | +0.82 *** | +0.33 ** | +0.30 * | +0.92 *** | +0.27 * |
| (10) Final perf. | +0.73 (0.06) |  |  |  |  |  |  |  |  |  | 1 | +0.60 *** | +0.82 *** | +0.31 * | +0.31 * | +0.92 *** | +0.29 * |
| (11) Cong. Rate | +0.65 (0.07) |  |  |  |  |  |  |  |  |  |  | 1 | +0.94 *** | -0.54 *** | +0.93 *** | +0.30 * | +0.10 |
| (12) Acc. : valid | +0.75 (0.08) |  |  |  |  |  |  |  |  |  |  |  | 1 | -0.21 | +0.75 *** | +0.59 *** | +0.16 |
| (13) Acc. : invalid | +0.68 (0.10) |  |  |  |  |  |  |  |  |  |  |  |  | 1 | -0.79 *** | +0.58 *** | +0.11 |
| (14) Criterion bias | +0.25 (0.46) |  |  |  |  |  |  |  |  |  |  |  |  |  | 1 | -0.00 | +0.06 |
| (15) d’ : cue sess | +1.23 (0.39) |  |  |  |  |  |  |  |  |  |  |  |  |  |  | 1 | +0.36 ** |
| (16) d’ : conf. sess | +1.21 (0.43) |  |  |  |  |  |  |  |  |  |  |  |  |  |  |  | 1 |

**Table S3: Descriptive statistics and Pearson correlations for all the variables. N= 65 participants. p-values:** *p<0.05; **p<0.01; ***p<0.001

The variables are: (1) Successful identification of the cues after the cueing session. (2) Ratio of Meta-d’ over d’. (3) Logarithm of this ratio. (4) resolution index from the Brier score. (5) average score in the working memory tests. (6) Calibrated difference in number of dots in the cueing session. (7) Order of the cueing and confidence session. (8) Initial performance in the cueing session. (9) Overall performance in the cueing session. (10) Final performance in the cueing session. (11) Rate of responses congruent with the predictive cue. (12) Accuracy in the valid cue trials. (13) Accuracy in the invalid cue trials. (14) Difference in the decision criteria for the left and right predictive cues. (15) d’ in the cueing session. (16) d’ in the confidence session.

**Figure S1. Individual data for the confidence session.** For each individual, the figure indicates the metacognitive efficiency (ME), the perceptual d’ and plots the distributions of response x confidence ratings (from high confidence left to high confidence right response) for the left stimulus (in blue) and the right stimulus (in green).

**SUPPLEMENTARY ANALYSES**

Mathematical argument

We propose here a formalization of our hypothesis linking metacognition to cue identification. We claim that the metacognitive ability of a participant, that is, her ability to assign a lower confidence to her errors, is positively related to her ability to identify the predictive values of the cues. This claim rests on the assumption that both metacognition and learning rely on how well the participant can process the information provided by the stimuli.

On a given trial, after receiving the stimulus ($S=R or L$) the observer would form a belief about the stimulus being *R*, noted $P(S=R)$, or *L*, noted $P(S=L)$. This belief will be the basis for the observer’s decision and confidence judgment: when $P\left( S=R \right)>P\left( S=L \right)$, the observer selects the response $R$ and her confidence is equal to $P\left( S=R \right)$, and when $P\left( S=R \right)<P\left( S=L \right),$she reports $L$ with confidence $P\left( S=L \right)$.

In a cue learning situation, this belief would be the basis for identifying the predictive value of the cue, that is, the probability $\pi$ of the stimulus $R$ when the cue is present. The observer can form an estimate $\hat{\pi}$ of this value by considering her average belief about the presence of the stimulus $R$ in these trials.

To formally relate $\hat{\pi}$ to the metacognitive ability of the observer, let us examine how it depends on the observer's average confidence when she is correct ($\bar{C_{C}}$) and her average confidence when she is incorrect ($\bar{C_{I}}$). As there are two types of stimuli and two types of responses, there will be 4 types of trials, described in the table below. Here, we also assume that the subject's accuracy rate $A$ and confidence when correct or incorrect do not depend on the stimulus being $R$ or $L$, for sake of simplicity.

| Stimulus presented  (with probability p) | Response made  (with probability p) | Confidence in the response | Belief about *R* being present |
| --- | --- | --- | --- |
| R, with p=$\pi$ | R, with p=$A$ | $\bar{C_{C}}$ | $\bar{C_{C}}$ |
|  | L, with p=$1-A$ | $\bar{C_{I}}$ | $1-\bar{C_{I}}$ |
| L, with p=$1-\pi$ | L, with p=$A$ | $\bar{C_{C}}$ | $1-\bar{C_{C}}$ |
|  | R, with p=$1-A$ | $\bar{C_{I}}$ | $\bar{C_{I}}$ |

Across these 4 cases, we can define the net belief that the stimulus $R$ was presented, that is, the estimate $\hat{\pi}$ made by the observer.

$$\hat{\pi}=\pi A\bar{C_{C}}+\pi(1-A)(1-\bar{C_{I}})+(1-\pi)A(1-\bar{C_{C}})+(1-\pi)(1-A)\bar{C_{I}}$$

Furthermore, if we assume that the observer’s mean confidence is equal to her accuracy rate $A$, we can produce a relation between $\hat{\pi}$, $A$ and the difference between $\bar{C_{C}}$ and $\bar{C_{I}}$, which we will denote by $r$ for the resolution of confidence with respect to errors. This difference quantifies metacognitive ability in a simplistic manner but it is appropriate for the present illustration.

The assumption that the average confidence is $A$ across all trials gives us: $A\bar{C_{C}}+\left( 1-A \right)\bar{C_{I}}=A$

By definition we also have $r=\bar{C_{C}}-\bar{C_{I}}$

Combining these two equations, we find that $\bar{C_{C}}=A-Ar+r$ and that $\bar{C_{I}}=A-Ar$

Note that the extreme case of perfect error detection would correspond to $r=1,\bar{C_{C}}=1,\bar{C_{I}}=0$ and that the extreme case of null resolution would correspond to $r=0,\bar{C_{C}}=A,\bar{C_{I}}=A$

Introducing these expressions in our calculation of $\hat{\pi}$ yields, after simplification:

$$\hat{\pi}=\pi-2A(2\pi-1)(1-A)(1-r)$$

Thus, for any given $\pi$ greater than 0.5, and assuming that both $A$ and $r$ are between 0 and 1, this expression tells us that $\hat{\pi}$ should always fall below $\pi$, and will get closer to $\pi$ when accuracy gets closer to 1 or when resolution gets closer to 1.

The figure below illustrates how $\hat{\pi}$ depends on *A* and *r*, for a target value of $\pi=0.75.$ We clearly see that the estimated probability of the cue-stimulus association approaches the target value of .75 (in red) when accuracy and confidence resolution increases. In other words, both perceptual ability and metacognitive ability improve cue identification.
