# Supplementary material for: Metacognitive ability predicts learning cue-stimulus associations in the absence of external feedback
Source: Sci Rep. 2018 Apr 4;8:5602. doi: 10.1038/s41598-018-23936-9 (PMC5884814; doi:10.1038/s41598-018-23936-9)
Supplement: Supplementary file 2 — Data description [file 41598_2018_23936_MOESM2_ESM.doc]

Data description for :

**Metacognitive ability predicts learning cue-stimulus associations in the absence of external feedback**

Marine Hainguerlot^1^, Jean-Christophe Vergnaud^1,*^, Vincent de Gardelle^2,*^

^1^Centre d’Economie de la Sorbonne, CNRS UMR 8174, Paris, France

^2^CNRS and Paris School of Economics, Paris, France.

^*^denotes equal contribution

Corresponding author: Vincent de Gardelle.

Centre d’Economie de la Sorbonne, 112 boulevard de l’Hopital, 75013 Paris, France

Tel: 0144078742. Email: [vincent.gardelle@gmail.com](mailto:vincent.gardelle@gmail.com)

Data contains 3 sheets: D, WM and C

D has the following fields:

.subject: the subject number.

.group: the experiment was run in 6 groups of participants.

.order: order of the 2 sessions: 1 for confidence first, 0 for cueing first.

.ntrials: trial number

.type_task: indicate the subpart of the experiment:

1, 2, 3 for the confidence session (training, calibration, main part)

4, 5, 6 for the cueing session (calibration, training, main part)

.cues: the cues used (in the cueing session): 1 for neutral, 2 for right, 3 for left

.stimulus: the stimulus category: 1 for left, 0 for right

.key: participant's response: 1 for left, 0 for right

.xc: number of points for the difference between left and right

.resp_acc: response accuracy

.resp_rt: response time

.conf_ans: the confidence answer on the rating scale (in the confidence session)

.conf_rt: the response time for the confidence scale

WM is a 65x3 matrix with subject number, and the 2 memory scores for each subject.

C is a 65x6 matrix indicating for each subject the identification of the cues. The first 3 columns indicate the responses of the participant for the left, right and neutral cues respectively. Here, 1 means neutral, 2 means right, 3 means left. The last 3 columns indicate whether the identication of the each cue (left, right and neutral) is correct or not.
